# Supplementary material for: Formation of Amino Acids and Carboxylic Acids in Weakly Reducing Planetary Atmospheres by Solar Energetic Particles from the Young Sun
Source: Life (Basel). 2023 Apr 28;13(5):1103. doi: 10.3390/life13051103 (PMC10221653; doi:10.3390/life13051103)
Supplement: Supplementary file 1 [file life-13-01103-s001.zip › Supplimetary Materials.pdf]

Supplimetary Materials

1. HPLC Chromatogram of authentic amino acid standards.

Figure S1 Shows ion-exchange HPLC chromatogram of authentic amino acid standards (WAKO ANII + B).

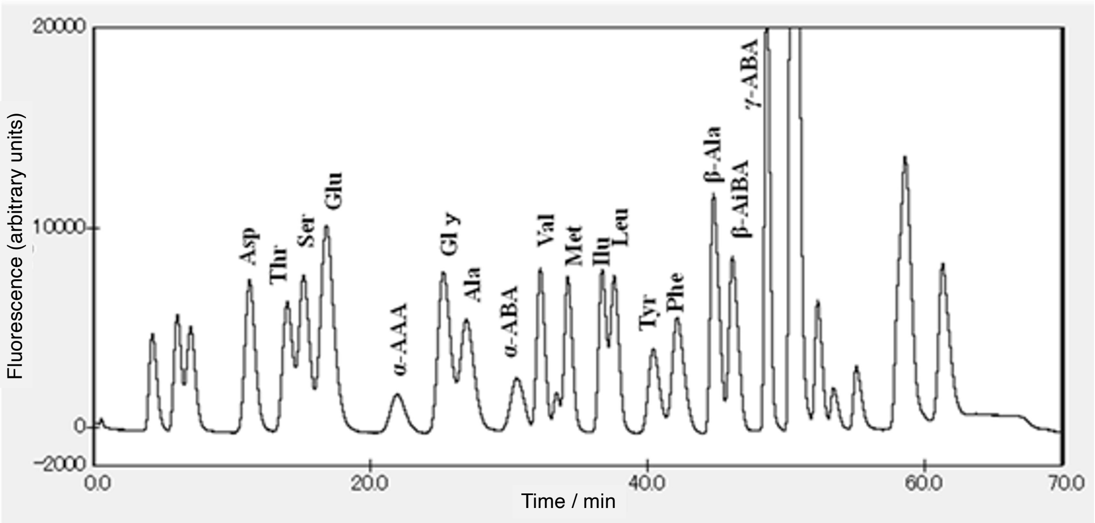

Figure S1. HPLC chromatogram of authentic amino acid standards.
AAA: aminoadipic acid, ABA: aminobutyric acid; AiBA: aminoisobutyric acid

2. Analysis of carboxylic acids by GC

Authentic standards were used to identify carboxylic acids by comparing the GC retention times. Monocarboxylic acids were derivatized to *p*-bromophenacyl esters [35] whereas dicarboxylic acids were derivatized to dibutyl esters [36]. As an example, a typical GC/FID trace is given below for the authentic standards of *p*-bromophenacyl ester derivatives of low molecular weight monocarboxylic acids (C_1-C_7). Figure S2 is a gas chromatogram of authentic monocarboxylic acids.

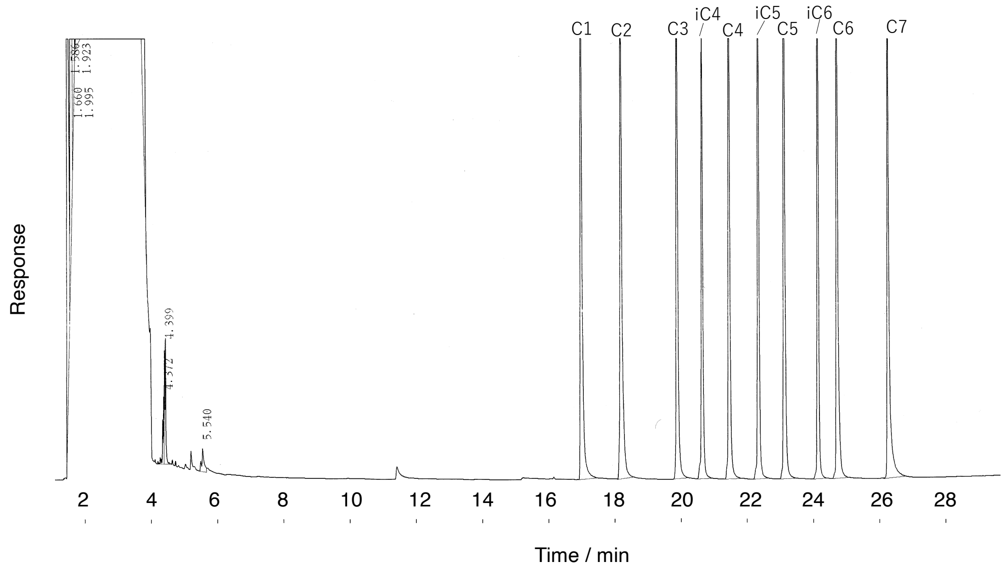

Figure S2. Capillary gas chromatogram of authentic standards of monocarboxylic acid (C_1–C_7) *p*-bromophenacyl esters. iC4 means isobutanoic acid or isobutyric acid (2-methylpropanoic acid). The same for iC_5 and other branched chain acids.
